# Geographical diet variations and microbial diversity: insights into François' langur’s adaptive strategies

**DOI:** 10.1128/msphere.00594-25

**Published:** 2026-02-24

**Authors:** Heqin Cao, Xiongwei Yang, Linzheng Hu, Qixian Zou, Guangrong Li, Wei Gou, Haijun Su

**Affiliations:** 1 Forestry College of Guizhou University, Guiyang, China; 2 Research Center for Bio-Diversity and Nature Conservation, Guizhou University 71206, Guiyang, China; 3 Aha Lake National Wetland Park Administration, Guiyang, China; 4 Guizhou Mayanghe National Nature Reserve Adminstration, Tongren, China; 5 Guizhou Kuankuoshui National Nature Reserve Administration, Zunyi, China; 6 Guizhou Dashahe National Nature Reserve Administration, Zunyi, China; University of South Africa, Florida, Johannesburg, Gauteng, South Africa

**Keywords:** *Trachypithecus francoisi*, diet composition, gut microbiota

## Abstract

The diversity in foraging behavior observed among species is indicative of their ability to adapt to specific environmental conditions, with dietary differences playing a crucial role in shaping the composition of gut microbiota. However, there are limited reports on the dietary habits and gut microbiota of François' langur (*Trachypithecus francoisi*) across different wild geographical populations. To address this, our study employed DNA metabarcoding and 16S rRNA sequencing to investigate variations in dietary composition and their influence on gut microbiota among distinct wild populations of François' langur, as well as among different groups within the same region. The dietary analysis revealed a broad diet, identifying 134 families and 336 genera of plants. The habitat quadrat survey results indicate significant differences in the habitats of François' langurs across different geographic populations. However, the dietary composition analysis reveals that while the food composition of different groups within the same region is relatively similar, there are notable differences across geographically distinct regions. The microbial community analysis demonstrated distinct compositional and structural divergence in gut microbiota between these populations, whereas no significant microbial differences were detected among groups within the same region. Further correlation analysis between diet and microbiota indicated that dominant plant taxa in the diet exhibited significant associations with Firmicutes, Proteobacteria, and other microbial phyla, displaying varying degrees of positive or negative correlations. This study elucidates how dietary variations among geographically distinct populations of François' langur drive changes in gut microbiota, reflecting their adaptive responses to local habitats. These findings provide valuable insights for the conservation management of François' langur populations and potential applications in health status monitoring.

**IMPORTANCE:**

Understanding the mechanisms by which animals adapt to their environment is essential for effective conservation efforts. This study examines the endangered François' langur, focusing on the largely unexplored relationship between its dietary habits and gut microbiota across various wild populations. Our research indicates that although habitat vegetation varies significantly even among groups within the same region, their diets remain similar. Conversely, langur populations from distinct geographic areas exhibit notable dietary differences. These dietary variations, in turn, lead to distinct compositional differences in their gut bacterial communities. This diet-microbiome interaction serves as a crucial physiological indicator of how these primates adapt to their local forest environments. By illustrating that gut microbiota composition reflects an animal’s ecological response to its environment, this study offers a powerful and non-invasive tool for conservation. These findings are critical for developing targeted strategies, such as habitat restoration, and for monitoring the health of these rare primates through gut microbiome analysis.

## INTRODUCTION

François' langur (*Trachypithecus francoisi*), belonging to Primates, Cercopithecidae, *Trachypithecus*, is a national priority protection wildlife in China. It is mainly distributed in fragmented karst mountain forests or river valleys in China (Guizhou province, Guangxi province, and Chongqing province), Vietnam, and Laos (1–6). The species was assessed as endangered (EN) on the IUCN Red List of Threatened Species in 2015 (criteria: A2acd+3cd; C1+2a(i)), with a global wild population estimated at 2,000–2,100 individuals (7), approximately 70% of the global François' langur population in Guizhou province. However, accelerated global industrialization and urbanization, coupled with anthropogenic habitat destruction, have led to continued range contraction and fragmentation of François' langur populations, resulting in numerous heterogeneous populations (4, 8, 9). In Guizhou province, five historical distribution sites have disappeared, leaving only five remote nature reserves: Mayanghe National Nature Reserve (herein referred to as MYH), Dashahe National Nature Reserve (herein referred to as DSH), Kuankuoshui National Nature Reserve, Baiqing Nature Reserve, and Yezhong Nature Reserve (10). Consequently, there is an urgent need for in-depth research on different geographical populations to develop effective conservation management strategies (11).

Foraging is a fundamental activity in animal survival, serving as the primary source of nutrients and energy required for growth, reproduction, and movement (12). Variability in foraging behavior is evident among species, groups, and individuals, reflecting diverse strategies shaped by natural selection and adaptation to local environments (13). Research has demonstrated that François' langurs exhibit a broad dietary range (at least 259 species of plants) and a notable preference for leaf consumption, mostly using the traditional instantaneous scan sampling method (14–16). However, traditional observation methods pose challenges in systematically tracking highly alert animals, leading to incomplete documentation of dietary items. The integration of high-throughput sequencing in animal diet research has enhanced the detail and scope of studies, aiding in the examination of wild animal diets (17). However, there is a scarcity of studies using DNA metabarcoding to analyze the diet of François' langur.

Emerging research suggests that gut microbiota significantly impacts disease susceptibility, nutritional status, immune function, and host maturation (18, 19). Conversely, environment and dietary habits are pivotal determinants of gut microbiota composition (20, 21). Numerous investigations have demonstrated that alterations in dietary intake and environmental conditions stemming from habitat modifications exert a notable impact on gut microbiota (22–24). Furthermore, research has indicated that host dietary patterns profoundly shape gut microbiota diversity (25), primarily through the provision of specific nutrients that promote microbial growth (26, 27). The ingestion of food leads to changes in the gut microbiota, metabolites, and short-chain fatty acids, affecting the metabolites generated by gut microbes and the biochemical processes within organisms. A decline or sustained decrease in the consumption of macronutrients, particularly fiber, may lead to the diminishment of essential microbiome communities (28–30). Studies have found that there is a considerable amount of heterogeneity among hosts of the same species, especially when those hosts live in geographically distinct populations (31, 32). Existing studies have mainly focused on captive populations, leaving a gap in the study of the gut microbiota of langurs in different geographical populations. So, further research is needed to determine if there are differences in gut microbiota among various populations of langurs in their natural habitats.

This study seeks to examine the impact of environmental and dietary factors on the gut microbial community of François' langur. Two distinct wild populations of François' langur were selected to address the following objectives: (i) assess potential differences in dietary composition of François' langur across varying environments; (ii) examine variations in gut microbiota composition and structure under different dietary conditions; (iii) explore the relationship between dietary patterns and gut microbiota. Utilizing metabarcoding and 16S sequencing technology, we comprehensively elucidate the dietary characteristics and gut microbiota profiles of François' langurs, thereby furnishing fundamental data for the conservation and management of this species.

## MATERIALS AND METHODS

### Study area and sample collection

This study was conducted in two national nature reserves (Mayanghe National Nature Reserve [herein referred to as MYH] and Dashahe National Nature Reserve [herein referred to as DSH]) in Guizhou Province, China. DSH is located in Zunyi city (107°21′52″–107°47′45″E, 29°00′20″–29°13′27″N), with a total area of 26,990 hm^2^, of which the total area of the core area is 9,104.0 hm^2^ (33.7% of the total area). This reserve primarily protects forest ecosystems and endangered species including silver fir (*Cathaya argrophylla*) and François' langur. MYH is located at the border of Tongren City and Zunyi City (108°03′48″–108°19′42″E, 28°37′26″–28°54′30″N), with a total area of 31,113 hm² and core area of 10,543 hm^2^ (33.9% of total area). This reserve focuses on protecting François' langur and its habitat.

**Fig 1:**
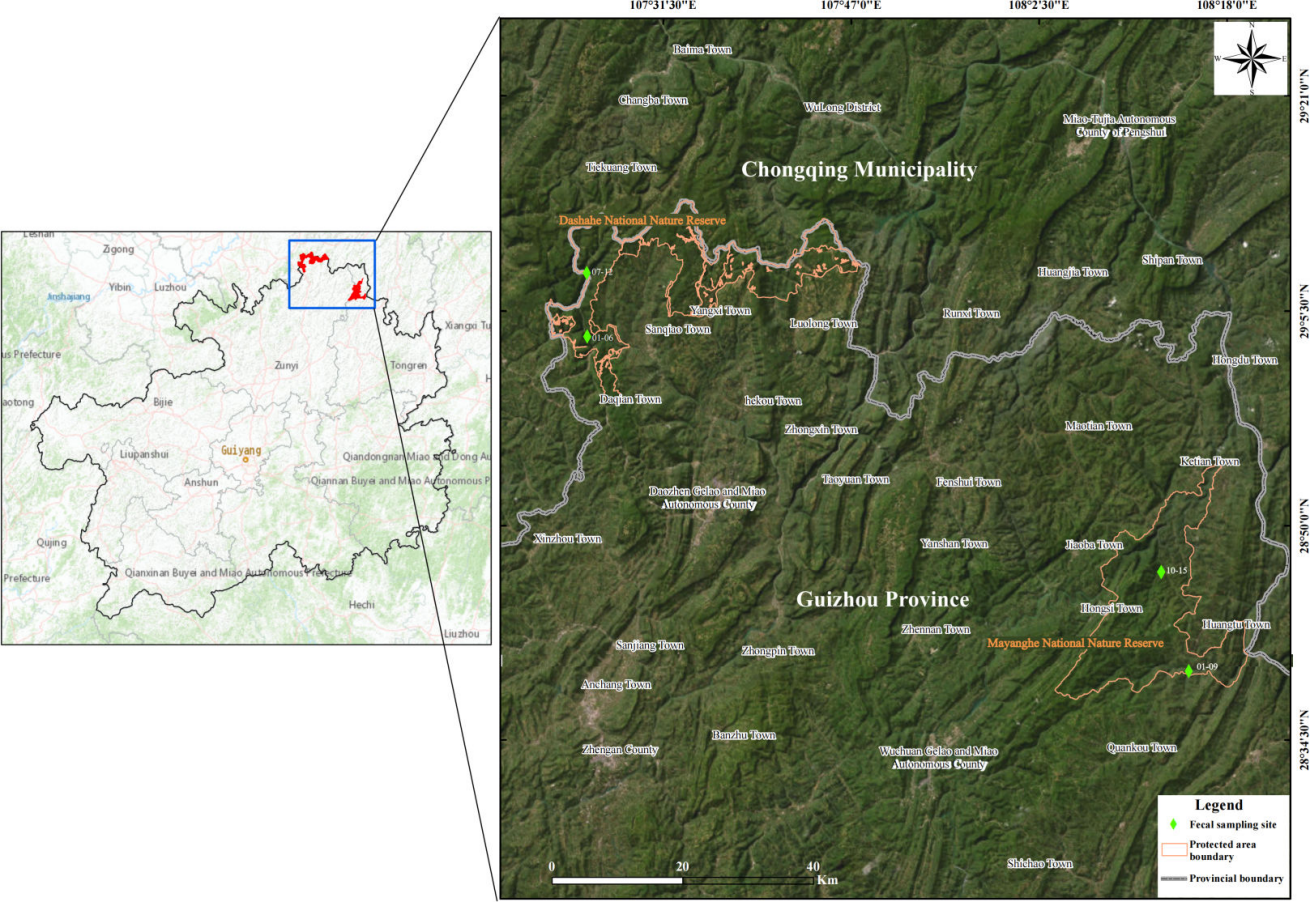
Fecal sampling sites of François' langurs.

In March 2022, 27 fresh fecal samples (<24 h) from four populations were collected from the night roosting sites of François' langurs in DSH (6 samples in DSH1 population; 6 samples in DSH2 population) and MYH (9 samples in MYH1 population; 6 samples in MYH2 population). Detailed information on fecal sample collection is provided in Table S1. Sampling was conducted during the same season to ensure synchronized plant phenology (Fig. 1). All samples were immediately transported to the laboratory on dry ice and stored at −80°C for subsequent analysis.

Two 10 m × 10 m vegetation quadrats were established near the fecal collection sites, recording the species names and abundances of the tree layer, shrubs, herbs, and interlayer vegetation (Table S2). Species richness index (R), Shannon-Wiener diversity index (H), and Pielou evenness index (E) were calculated to characterize the alpha diversity of the community. The Sørenson similarity index (C_S_) was used to represent the differences between the two regions and groups (33). The quadrat results are presented in Table 1. The alpha diversity indices of MYH were consistently higher than those of the DSH region, and the similarity indices were relatively low both between different regions and among quadrats from different monkey groups within the same region.

**TABLE 1:** Plant diversity indices based on quadrat data^*a*^

Group	*R*	*H*	*E*	*C*_*S*_
DSH	43	3.23	0.86	0.185
MYH	65	3.64	0.87	
DSH1	21	2.54	0.83	0.089
DSH2	24	2.63	0.83	
MYH1	32	3.04	0.88	0.088
MYH2	36	3.08	0.86	

^*a*^ *R* is species richness index, *H* is Shannon-Wiener diversity index, *E* is Pielou evenness index, and *C*_*S*_ is Sørenson similarity index.

The calculation formulas are as follows:


R=S

$$H=-\sum_{i=1}^{S}p_{i}ln\;p_{i}$$

$$E=\frac{H}{ln\;S}$$

$$C_{s}=\frac{2c}{a+b}$$

where *S* represents the number of species identified within the quadrat, *P_i_* denotes the proportion of the *i*th species relative to the total number, *a* and *b* indicate the number of species in any two groups, and *c* signifies the number of species common to both groups.

### DNA extraction and PCR amplification

Total genomic DNA samples were extracted using the OMEGA Soil DNA Kit (M5635-02) (Omega Bio-Tek, Norcross, GA, USA), following the manufacturer’s instructions, and stored at −20°C prior to further analysis. The quantity and quality of extracted DNAs were measured using a NanoDrop NC2000 spectrophotometer (Thermo Fisher Scientific, Waltham, MA, USA) and agarose gel electrophoresis, respectively.

Sample-specific 7-bp barcodes were incorporated into the primers for multiplex sequencing. The PCR components contained 5 μL of buffer (5×), 0.25 μL of Fast pfu DNA Polymerase (5U/μL), 2 μL (2.5 mM) of dNTPs, 1 μL (10 µM) of each Forward and Reverse primer, 1 μL of DNA Template, and 14.75 μL of ddH2O. Thermal cycling consisted of initial denaturation at 98°C for 5 min, followed by 25 cycles consisting of denaturation at 98°C for 30 s, annealing at 53°C for 30 s, and extension at 72°C for 45 s, with a final extension of 5 min at 72°C. PCR amplicons were purified with Vazyme VAHTSTM DNA Clean Beads (Vazyme, Nanjing, China) and quantified using the Quant-iT PicoGreen dsDNA Assay Kit (Invitrogen, Carlsbad, CA, USA). After the individual quantification step, amplicons were pooled in equal amounts, and pair-end 2*250 bp sequencing was performed using the Illumina NovaSeq platform with NovaSeq 6000 SP Reagent Kit (500 cycles) at Shanghai Personal Biotechnology Co., Ltd (Shanghai, China).

PCR amplification of the bacterial 16S rRNA genes V3–V4 region was performed using the forward primer 338F (5′-ACTCCTACGGGAGGCAGCA-3′) and 806R (5′-GGACTACHVGGGTWTCTAAT-3′). PCR amplification of chloroplast rbcL gene was performed using the forward primer Z1aF (5′-ATGTCACCACCAACAGAGACTAAAGC-3′) and hp2R (5′-CGTCCTTTGTAACGATCAAG-3′).

### Sequence analysis

Microbiome bioinformatics were performed with QIIME2 2019.4 (34) with slight modification according to the official tutorials. Raw sequence data were demultiplexed using the demux plugin followed by primers cutting with cutadapt plugin. Sequences were then quality filtered, denoised, merged, and chimera removed using the DADA2 plugin (35). The DNA sequences were compared in the online databases of NCBI (https://blast.ncbi.nlm.nih.gov/ accessed on 07 June 2022) and BOLDsystem (http://www.boldsystems.org/ accessed on 08 June 2022) to ensure that the obtained sequences were included in the diet of François' langur (refer to results of Li [14] and comprehensive scientific expedition data of DSH and MYH). The criteria for species identification were as follows: When the consistency of comparison results is higher than 97%, the most matched sequence only corresponds to a single plant species, and when the species is distributed locally, the sequence is considered to be from this species. If the most matched sequence corresponds to multiple plant species, it is necessary to exclude species that are not distributed locally, and if it still corresponds to more than one species, the identification result contains the minimum taxon of these species. The entire experimental process above was performed by Shanghai Personal Biotechnology Co., Ltd.

### Bioinformatics and statistical analysis

We used relative read abundance (RRA) to estimate the dietary composition and microbes of François' langur. RRA is the percentage of the sequence number of a certain diet/microbe category in the total diet/microbe sequence of the sample, which reflects the relative biomass, and the calculation formula is as follows:


$$RRA_{i}=\frac{1}{N}\sum_{j=1}^{N}\frac{S_{ij}}{\sum_{i=1}^{T}S_{ij}}\times 100\%$$

Where *T* is the number of diet/microbe categories, *N* is the total number of valid samples, and *S_i_*_,_*_j_* is the sequence number of diet/microbe category *i* in sample *j*. The sum of the RRAs of all diet/microbe groups is 100%.

The rank abundance curve was generated using R software to assess the rationality and adequacy of the sequencing data. Taxonomic abundance was represented as the mean ± standard deviation (SD) and visualized through histograms utilizing R version 4.2.2. Alpha diversity indices, including Chao1, Good’s coverage, Simpson, Pielou’s evenness, Shannon, and observed species, were calculated using Mothur version 1.30 and depicted via box plots. A Venn diagram was generated to visualize the shared and unique OTUs among samples or groups using R package “VennDiagram” based on the occurrence of OTUs across samples/groups regardless of their relative abundance.

Inter-group variations in alpha diversity were examined utilizing the Kruskal-Wallis test. Beta diversity was evaluated through Bray-Curtis distances, with the findings illustrated via non-metric multidimensional scaling (NMDS) analysis plots. Significant inter-group differences were tested using permutational multivariate analysis of variance (PERMANOVA) based on Bray-Curtis distances. To further investigate the differences in diet/microbes composition between MYH and DSH, the LEFSe (linear discriminant analysis effect size) analysis was performed with a LDA threshold of 4. Then, we used a random forest classifier to assess the predictive power of genus levels for different area and groups. Spearman correlations were computed in R using the cor() function. Network analysis was conducted with the igraph package, and visualizations were generated using ggplot2. By analyzing the differences of KEGG metabolic pathways using PICRUSt2 analysis, we can detect differences in metabolic pathways of functional microbial genes between MYH and DSH. Statistical comparisons were performed using the Statistical Package for the Social Sciences (SPSS, version 22.0, Chicago, USA). A *P*-value of less than 0.05 was considered indicative of statistical significance.

## RESULTS

### Diet analysis of François' langur

#### Diet diversity analysis of François' langur

**Fig 2:**
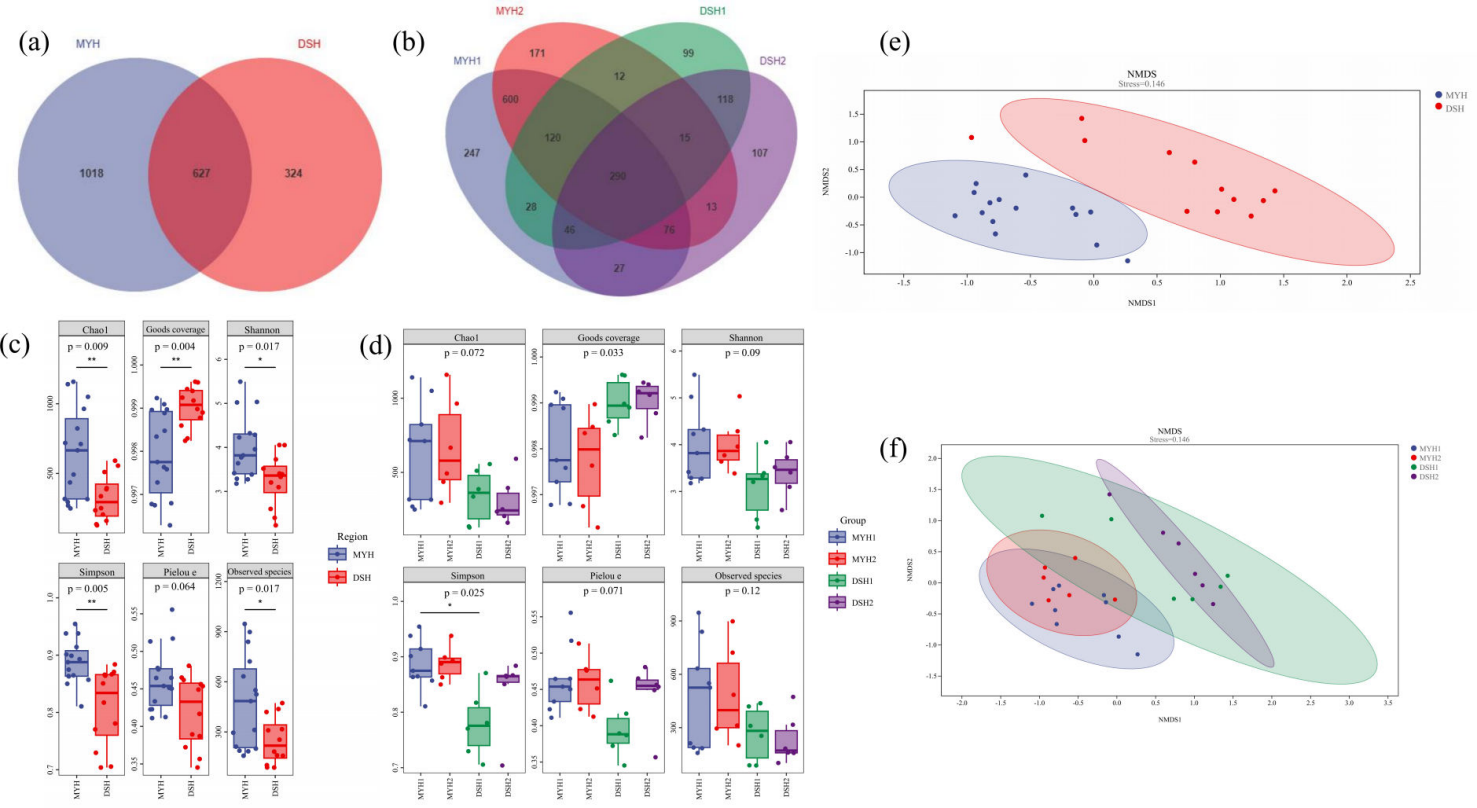
Diversity analysis of François' langur in MYH (Mayanghe National Nature Reserve) and DSH (Dashahe National Nature Reserve). Venn diagram for dietary OTU compositions in DSH and MYH (**a**) and four groups (**b**). Alpha diversity of François' langur in DSH and MYH (**c**) and four groups (**d**) (***P < 0.001, **0.001 < *P* < 0.01, *0.01 < *P* < 0.05). NMDS analysis of François' langur in DSH and MYH (**e**) and four groups (**f**).

A total of 6,431,715 original sequences and 5,738,654 effective sequences were obtained from the 27 fecal content samples of François’ langurs by means of amplicon chloroplast rbcL gene. Overall, 1,969 OTUs were identified from all samples. Venn diagram analysis identified 627 OTUs shared between MYH and DSH (Fig. 2a) with 290 OTUs common to all 4 groups (Fig. 2b).

The analysis of alpha diversity in dietary composition across different regions revealed significant differences between MYH and DSH in terms of Chao 1 (*P* < 0.01), Goods coverage (*P* < 0.01), Shannon indices (*P* < 0.05), Simpson (*P* < 0.01), and Observer species (*P* < 0.05) (Fig. 2d). The PCoA analysis results showed there were significant differences in the diet composition of François' langurs between MYH and DSH (Fig. 2c and h). The results of the PERMANOVA based on Bray-Curtis distance indicate significant differences between the DSH and MYH of François' langurs (PERMANOVA: Pseudo-*F* = 12.551, *R*^2^ = 0.207, *P* = 0.001).

The alpha diversity analysis (Fig. 2e) in dietary composition in different groups within the same geographic region revealed no significant differences. However, significant variations in Goods coverage (*P* < 0.05) and Simpson index (*P* < 0.01) were observed among groups from different regions. Beta diversity analysis showed significant difference in four groups (Fig. 2f and g). Based on Bray-Curtis distances demonstrated that PERMANOVA detected no significant dietary differences between groups within the same region, whereas inter-regional comparisons showed pronounced compositional divergence (PERMANOVA: Pseudo-*F* = 3.782, *R*^2^ = 0.330，*P* = 0.001).

#### Diet composition in different regions and different groups of François' langur

**Fig 3:**
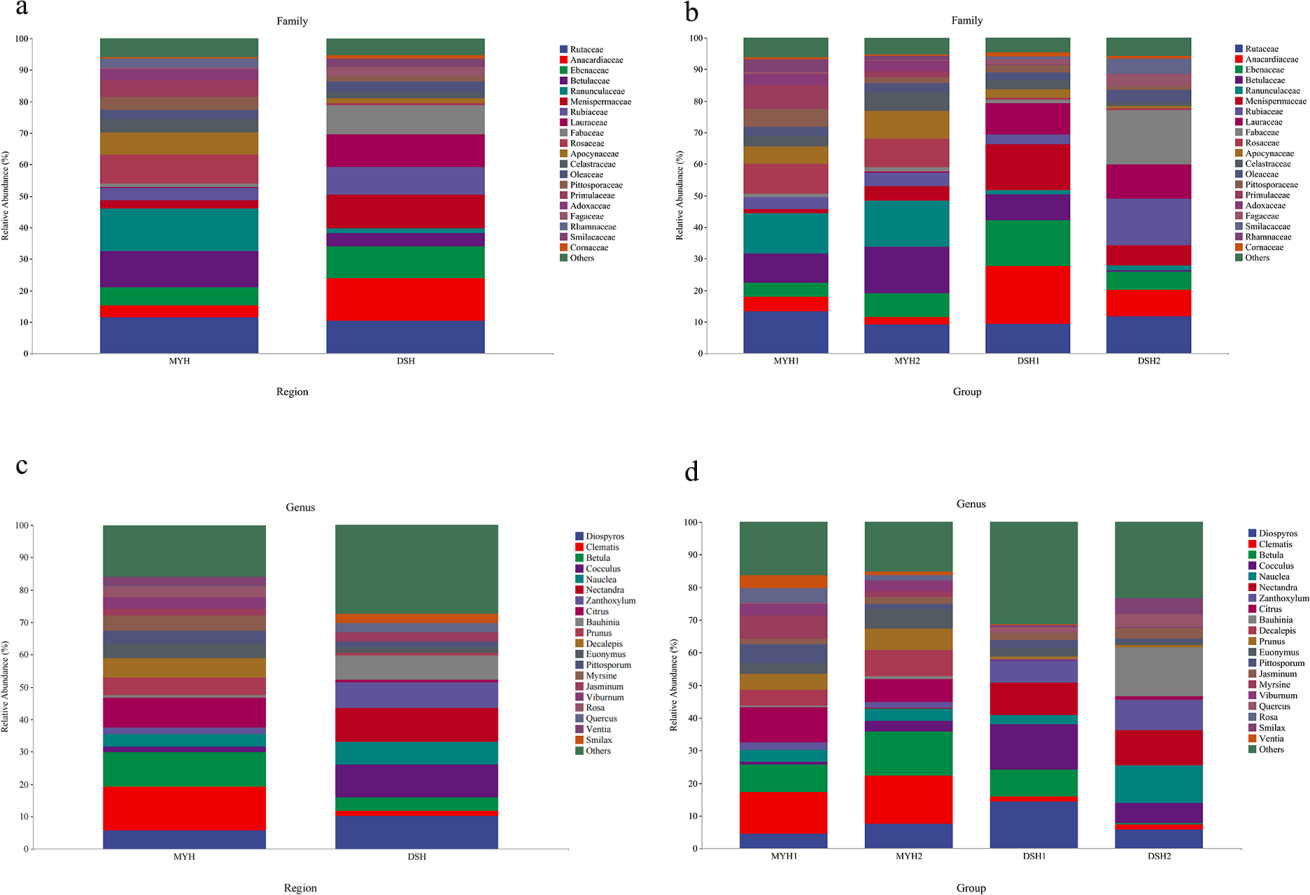
Relative dietary abundance at the family (**a and b**) and genus (**c and d**) level of François' langur in MYH and DSH.

**Fig 4:**
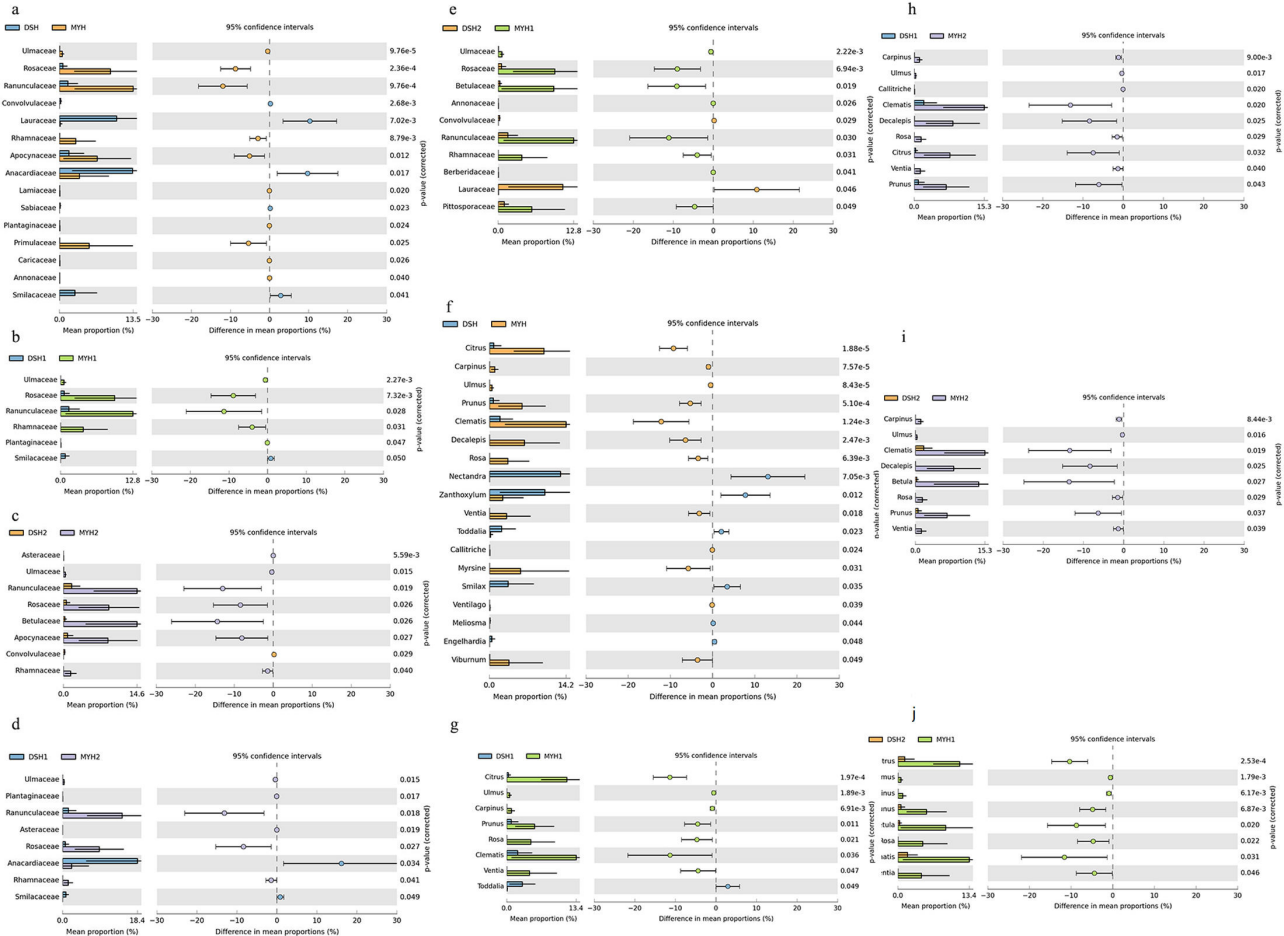
Comparison of relative dietary abundances at the family (**a–e**) and genus (**f–j**) level of François' langur.

A total of 134 families were identified in the diet composition of François' langur in MYH and DSH, and there were 10 common families, with relative abundances exceeding 1%. The dietary composition of François' langur was dominated by Ranunculaceae (13.52% ± 10.76%), Rutaceae (11.64% ± 6.88%), Betulaceae (11.49% ± 9.78%), and Rosaceae (9.34% ± 6.67%) in MYH, whereas they were dominated by Anacardiaceae (13.39% ± 11.09%), Rutaceae (10.51% ± 8.54%), Lauraceae (10.47% ± 10.31%), Menispermaceae (10.42% ± 13.57%), and Ebenaceae (10.19% ± 15.79%) in DSH (Fig. 3a). The Wilcoxon rank-sum test revealed that the relative abundance of Anacardiaceae (*P*<0.001) and Lauraceae (*P*<0.001) at the family level was significantly higher in the DSH than in the MYH. In contrast, the relative abundance of five families, including Rosaceae (*P* < 0.001), Ranunculaceae (*P* < 0.001), Apocynaceae (*P* < 0.001), Celastraceae (*P* = 0.002), and Primulaceae (*P* < 0.001) was significantly higher in the MYH than in the DSH (Fig. 4a).

At the genus level, 336 genera were identified in the diet composition of François' langur in MYH and DSH. The dominant taxa in MYH were *Clematis* (12.14% ± 12.60%), *Prunus* (11.52% ± 20.15%), *Betula* (10.60% ± 11.11%), *Citrus* (9.14% ± 8.44%), and *Diospyros* (7.80% ± 17.13%), whereas *Nectandra* (11.20% ± 9.31%), *Zanthoxylum* (8.64% ± 6.12%), *Betula* (7.33% ± 14.20%), *Cocculus* (6.75% ± 10.83%), and *Diospyros* (6.70% ± 12.49%) were the dominant genera in DSH (Fig. 3b). *Nectandra* (*P* < 0.001), *Zanthoxylum* (*P* < 0.001), and Cocculus (*P* = 0.01) showed higher abundance in the DSH than in the MYH. Moreover, the proportion of *Decalepis* (*P* < 0.001), *Clematis* (*P* = 0.008), *Citrus* (*P* < 0.001), and *Myrsine* (*P* < 0.001) was higher in the MYH than in the DSH (Fig. 4b).

#### Diet difference of François' langur in different areas

**Fig 5:**
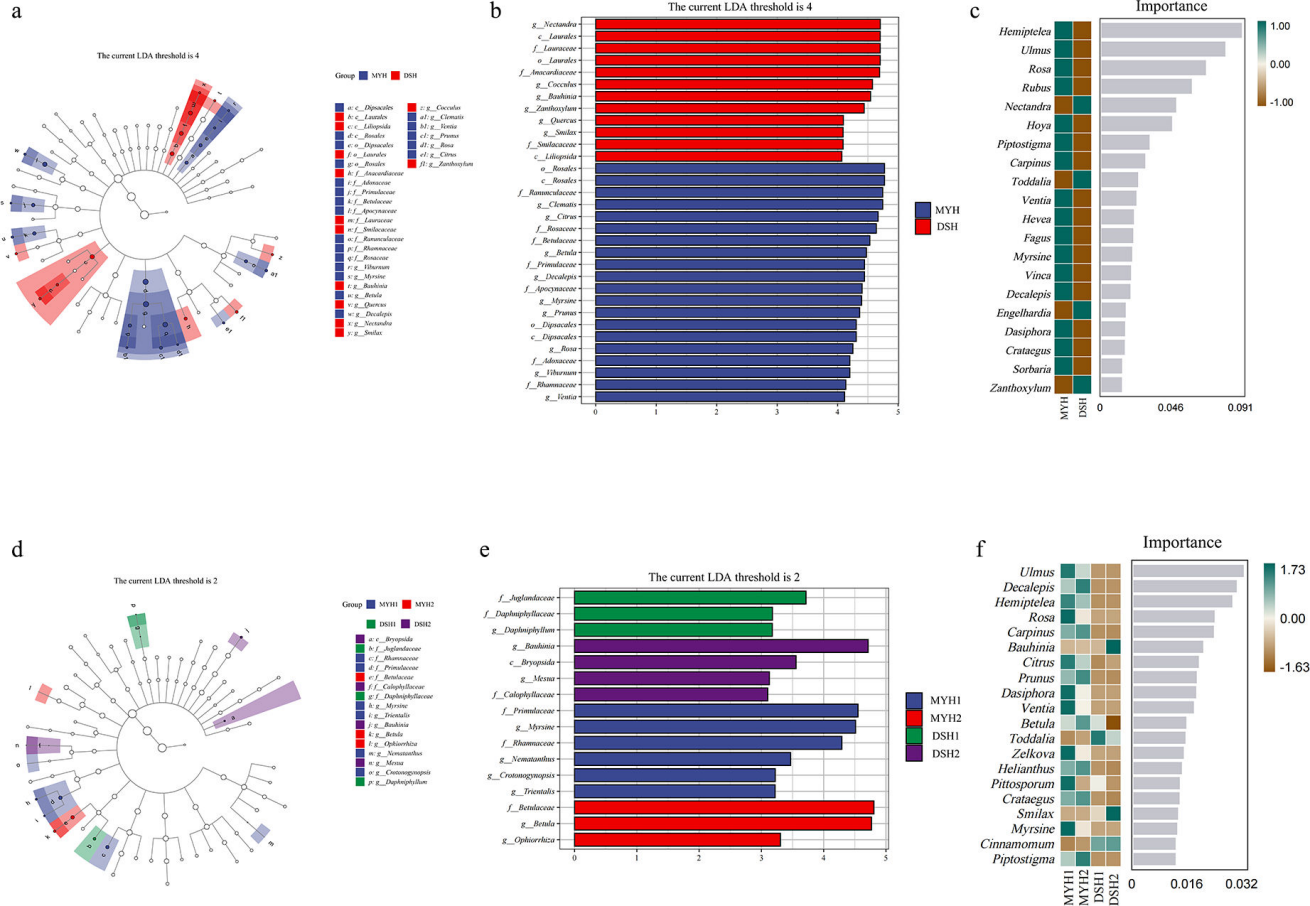
LEfSe analysis of diet composition differences of François' langur in different areas (**a, b**) and in different groups (**d, e**). The circles radiating from inside to outside represent taxonomic levels from phylum to genus, and colored nodes indicate significant differences in different areas (MYH [Mayanghe National Nature Reserve] and DSH [Dashahe National Nature Reserve]) and different groups. Additionally, plots illustrating the importance of random forest variables are presented at the genus level in different areas (**c**) and in different groups (**f**).

The LEfSe results revealed that a total of 37 biomarkers with statistical differences (9 in DSH, 28 in MYH). The significant biomarkers between MYH and DSH were distributed in Rosales (18.91%), Sapindales (16.22%), and Ericales (13.51%) (Fig. 5a and b). Furthermore, the results of the random forest revealed that among the taxa that differed between the MYH and DSH, the greatest contribution was made by Ulmaceae at the family level (AUC_MYH_ = 100.0000, AUC_DSH_ = 92.8571) and *Hemiptelea* at the genus level (AUC_MYH_ = 100.0000, AUC_DSH_ = 78.5714) (Fig. 5c and d).

### Gut microbiota of François' langur

#### Microbial diversity of François' langur

**Fig 6:**
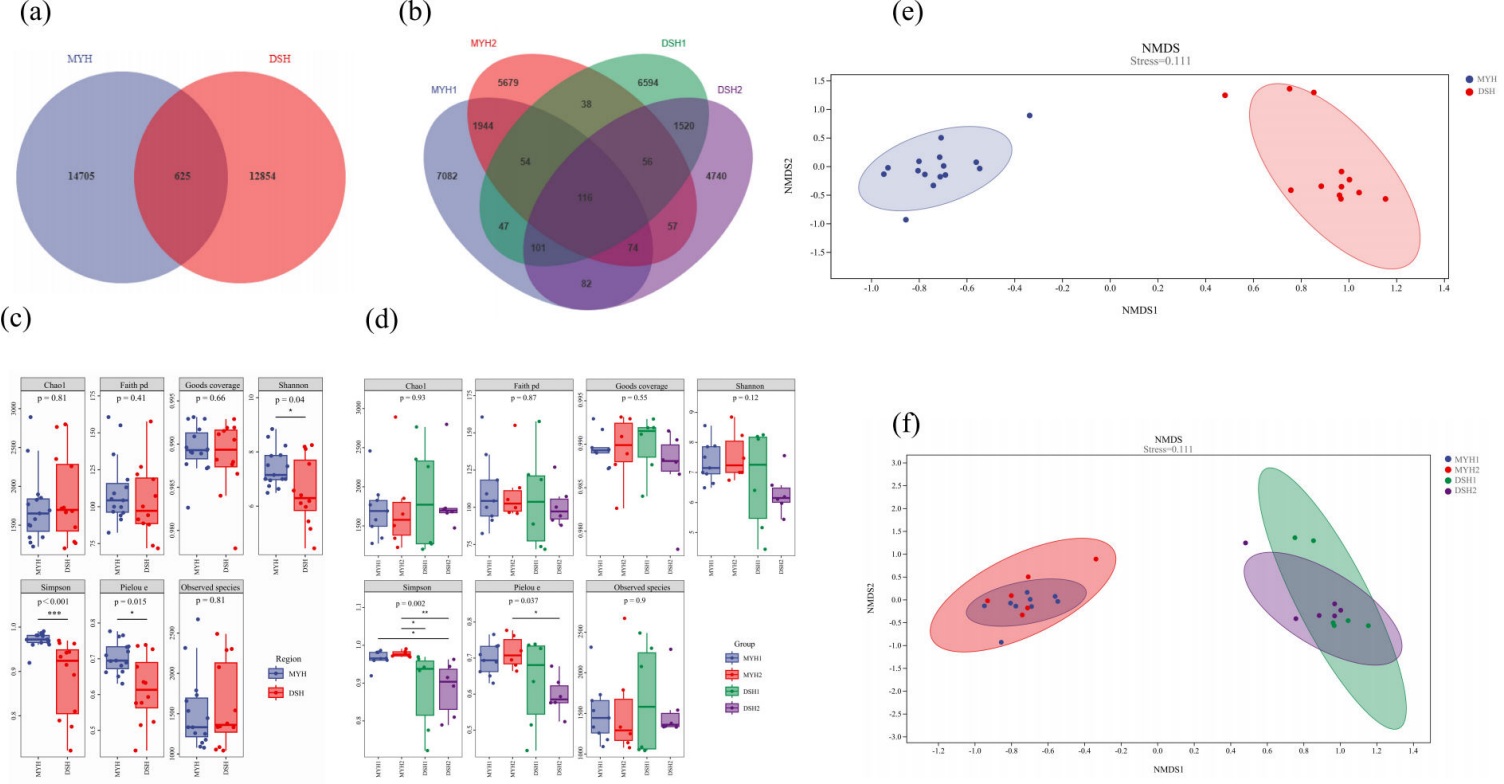
Microbial diversity analysis of François' langurs. Venn diagram for microbiota OTU compositions in MYH (Mayanghe National Nature Reserve) and DSH (Dashahe National Nature Reserve) (**a**) and different groups (**b**). Alpha diversity in DSH and MYH (**c**) and different groups (**d**), ***P < 0.001, **P < 0.01, *0.01 < *P* < 0.05. NMDS in DSH and MYH (**e**) and different groups (**f**).

A total of 67,313,742 original sequences and 5,966,530 effective sequences were obtained from the 62 fecal microbiota of François' langur using 16S sequencing analysis. Overall, 51,568 OTUs were identified from all samples analyzed. Venn analysis showed that there are 1,987 OTUs shared by MYH and DSH (Fig. 6a).

Alpha diversity analysis showed that there were extremely significant differences in Chao 1 (*P* < 0.05), Simpson (*P* < 0.01), and Pielou_e (*P* < 0.05) between MYH and DSH (Fig. 6b). Also, the NMDS analysis results showed there were significant differences in the microbial composition of François' langur between MYH and DSH (Fig. 6c and d). The results of the PERMANOVA based on Bray-Curtis distance indicate significant differences between the DSH and MYH of François' langurs (PERMANOVA: Pseudo-*F* = 8.125, *R*^2^ = 0.119，*P* = 0.001).

#### Fecal microbiota composition of François' langur in different areas

**Fig 7:**
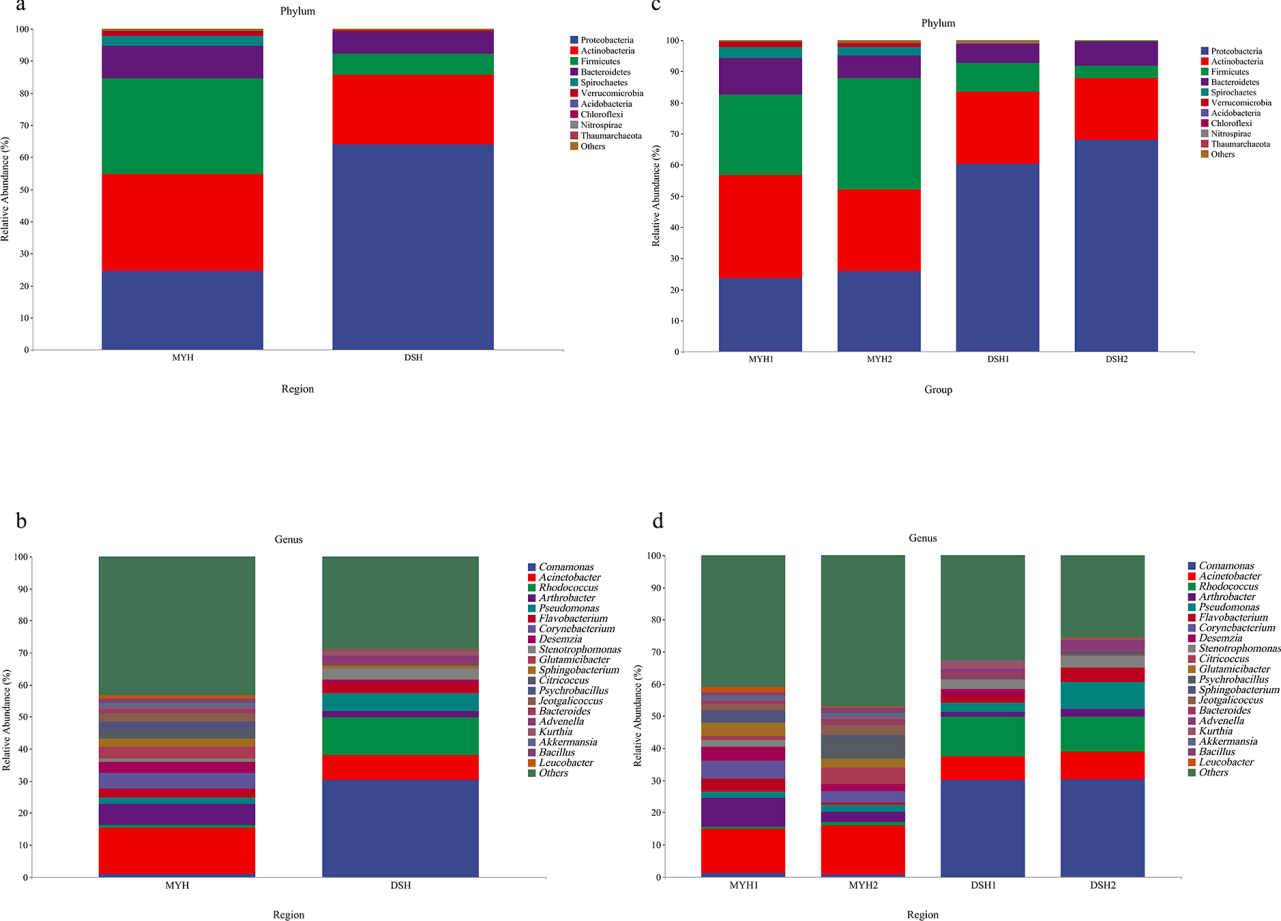
Relative gut microbiota abundance at the phylum (**a, c**) and genus (**b, d**) level of François' langur in MYH (Mayanghe National Nature Reserve) and DSH (Dashahe National Nature Reserve).

A total of 22 phyla were identified in the microbial composition of François' langur in MYH and DSH. Firmicutes (MYH: 32.07% ± 22.02%; DSH: 43.96% ± 41.16%), Actinobacteria (MYH: 31.32% ± 22.01%; DSH: 13.95% ± 22.25%), Proteobacteria (MYH: 25.13% ± 16.39%; DSH: 34.15% ± 34.87%) were the main components of François' langur in MYH and DSH (Fig. 7). At the genus level, *Acinetobacter* (15.93% ± 15.15%), *Arthrobacter* (4.99% ± 5.76%), and *Corynebacterium* (4.48% ± 8.26%) were the dominant genera in MYH. Whereas the dominant taxa were *Comamonas* (14.84% ± 22.68%), *Desemzia* (12.97% ± 22.96%), and *Rhodococcus* (5.64% ± 13.42%) in DSH (Fig. 7). There were seven common dominant genera of both groups, including *Desemzia*, *Rhodococcus*, *Acinetobacter*, *Pseudomonas*, *Akkermansia*, *Flavobacterium,* and *Ruminococcus*.

**Fig 8:**
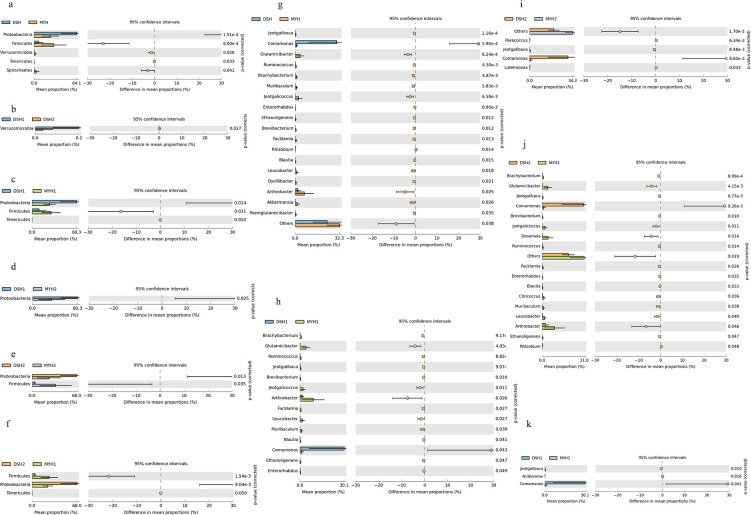
Comparison of relative abundances at the phylum and the genus level of François' langur in different regions (**a and g**) and different groups (**b–k**).

The Wilcoxon rank-sum test revealed that the relative abundance of Actinobacteria (*P* = 0.0009) and Spirochaetes (*P* = 0.002) at the phylum level was significantly higher in the MYH than in the DSH (Fig. 8a). Also, the relative abundance of these genera, including *Acinetobacter* (*P* < 0.001), *Arthrobacter* (*P* < 0.001), *Corynebacterium* (*P* < 0.001), *Glutamicibacter* (*P* < 0.001), *Jeotgalicoccus* (*P* < 0.001), *Citricoccus* (*P* = 0.024), and *Sphingobacterium* (*P* = 0.020) at the genus level in MYH were all significantly higher than those in DSH (Fig. 8b).

#### Differences in fecal microbial composition of François' langur in different areas

**Fig 9:**
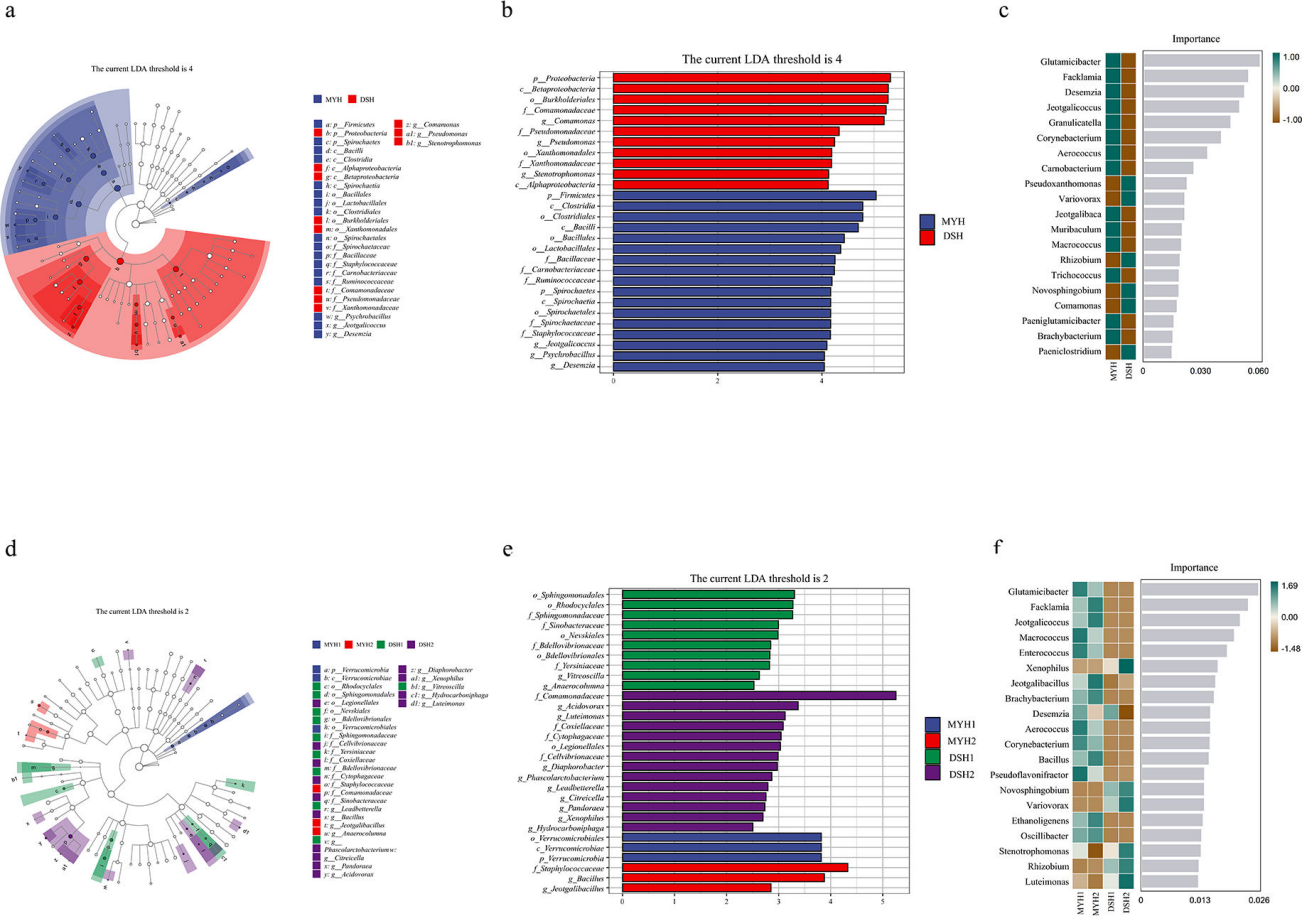
LEfSe analysis (**a, b, d, e**) of microbial composition differences of François' langurs. The circles radiating from inside to outside represent taxonomic levels from phylum to genus, and colored nodes represent there were significant differences in different areas and groups. Plots of results for the importance of random forest variables in different areas (**c**) and groups (**f**) at genus level.

The LEfSe analysis results revealed that a total of 28 biomarkers with statistical differences between MYH and DSH (17 in MYH, 11 in DSH) and 30 biomarkers in four groups (3 in MYH1, 3 in MYH1, 10 in DSH1, 14 in DSH2). The most significant biomarkers between MYH and DSH were distributed in Firmicutes (46.43%) and Proteobacteria (35.71%). In four groups, the most significant biomarkers were distributed in Proteobacteria (46.67%) and Firmicutes (23.33%). Furthermore, we used a random forest classifier to assess the predictive power of genus levels for different area and groups. We obtained the 20 most important genera that distinguish different regions and different populations of François' langur. It was found that *Glutamicibacter* and *Facklamia* were specifically and significantly indicative of MYH. *Pseudoxanthomonas* and *Variovorax* were of higher relative importance in DSH (Fig. 9c and f).

#### PICRUSt function prediction

**Fig 10:**
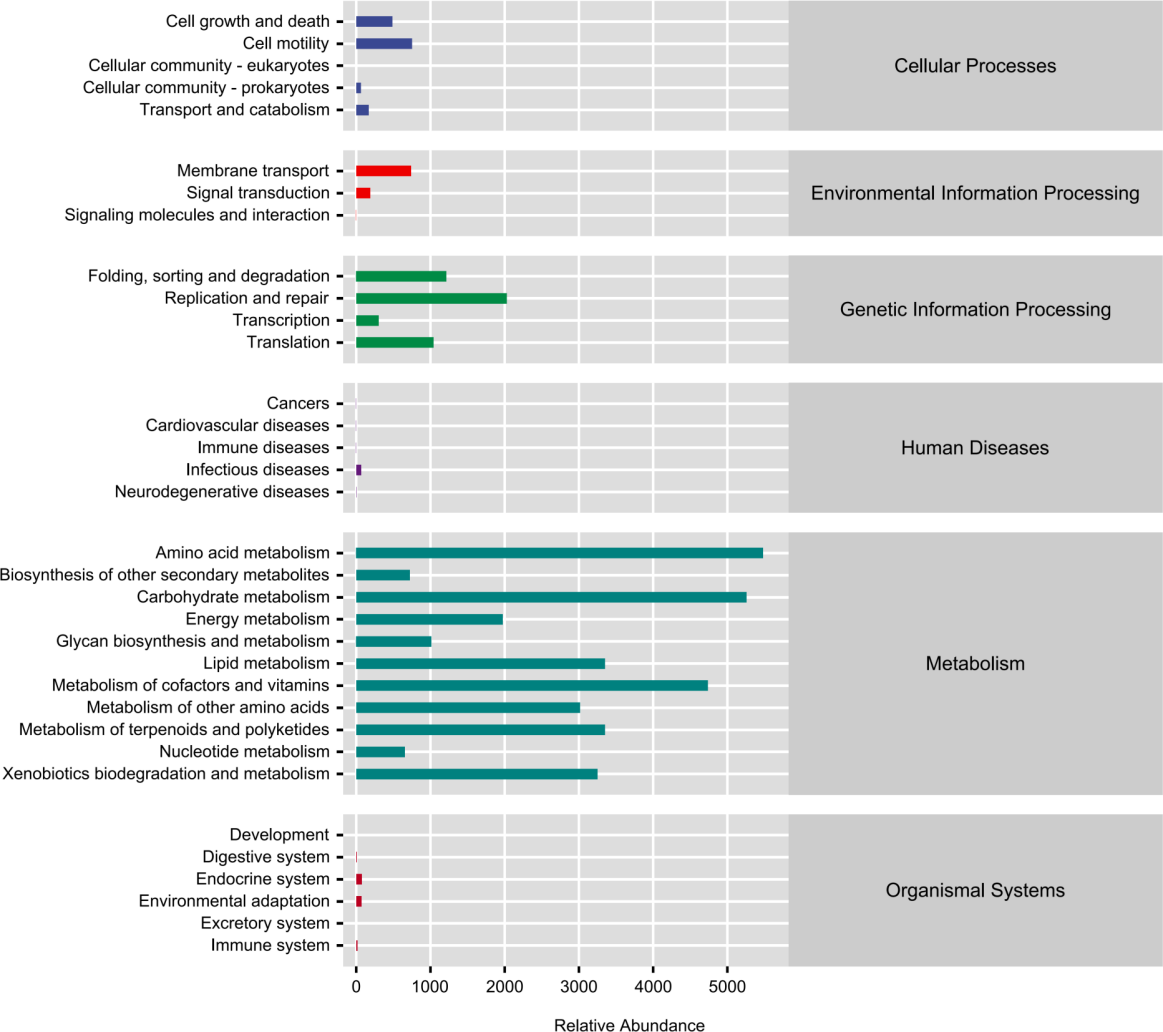
KEGG metabolic pathways.

A total of 34 KEGG metabolic pathways were analyzed (Fig. 10). The results showed that the metabolic pathways involved in metabolism had the highest abundance. Amino acid biosynthesis, carbohydrate metabolism, and metabolism of cofactors and vitamins were the five highest metabolic pathways in MYH and DSH (Fig. 10). Also, there were significant differences in 26 metabolic pathways (Table S3). A highly significant difference was observed in the abundance of the arachidonic acid metabolism (ko00590) (*P* = 0.0028) and zeatin biosynthesis (ko00908) (*P* = 0.0056) pathways between MYH and DSH.

### Correlation of gut microbiota with diet composition of François' langur

**Fig 11:**
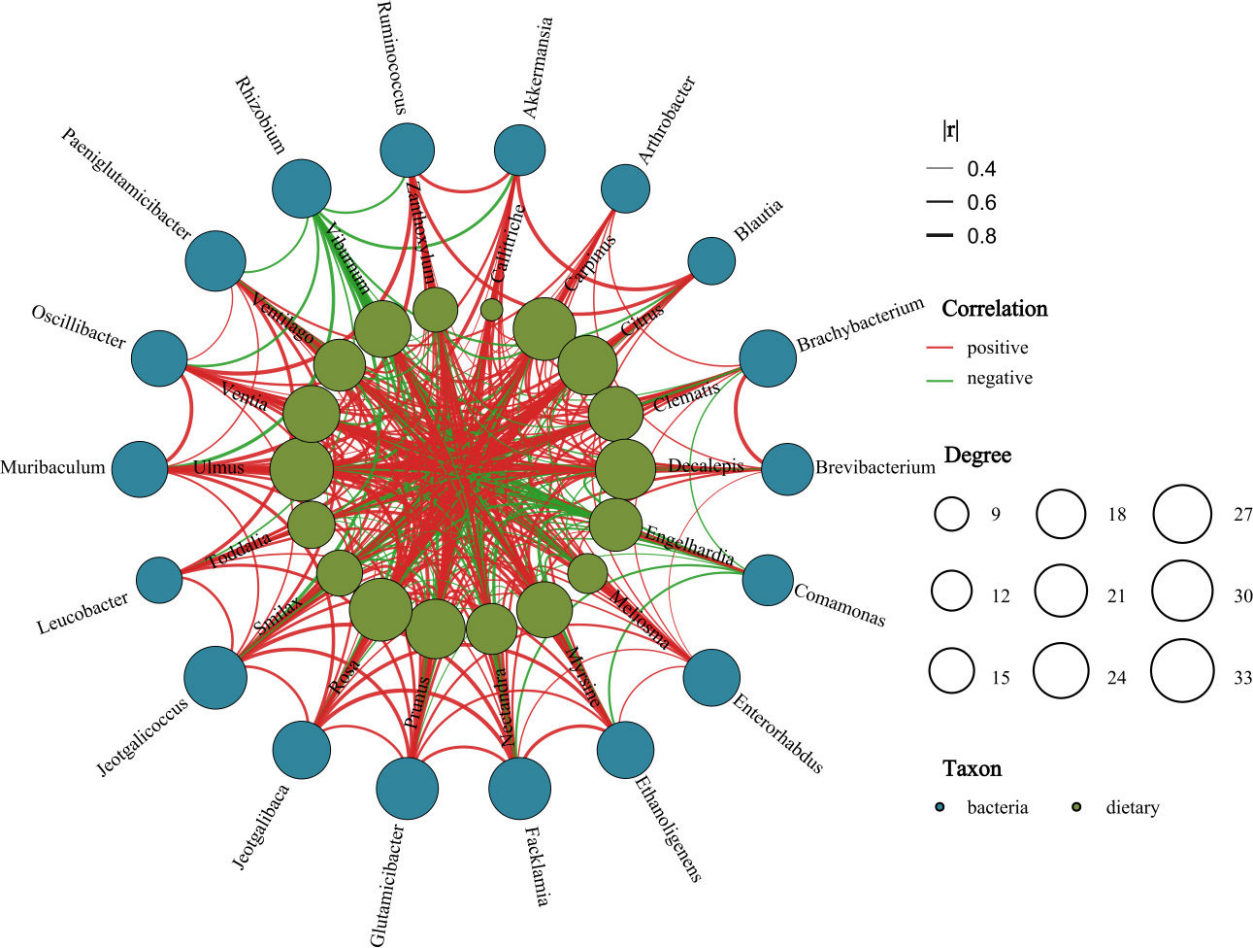
Relationships between significantly different diet composition and abundant microbes at the genus level of the François' langur in different wild populations. A connection indicates that the microbes have a correlation with the diet composition, a red solid line indicates a positive correlation, a green line indicates a negative correlation, and the size of the circle indicates the degree of the correlation.

Spearman correlation analysis of significantly different diet composition and significantly different microbes was performed to obtain the relationships between diet and microbes (Fig. 11). In the overall network diagram, the dominant genera of food composition were most closely related to the bacteria in Firmicutes and Proteobacteria showing different positive or negative correlations. Most dietary were significantly positively correlated with almost all gut bacteria, such as *Nectandra, Carpinu*s, *Ulmus*, *Meliosma*, and *Toddalia,* whereas *Engelhardia*, *Rhizobium,* and *Smilax* were significantly negatively associated with gut bacteria. In summary, the composition of the gut microbiota of François' langur in DSH and MYH was significantly different, as was the diet composition and structure. Changes in the composition of food changed the structure of intestinal flora to some extent.

## DISCUSSION

This study represents a novel exploration into the structural and functional disparities in gut microbiota and dietary habits of Francois' langurs, as well as the relationship between diet and gut microbiota in different wild populations through the utilization of metabarcoding and fecal 16s sequencing techniques. Our findings highlight the critical role of dietary patterns and environment in shaping gut microbial diversity and composition, revealing significant variations in both diet and microbiota between populations inhabiting different karst ecosystems in Guizhou Province, China.

### Diet composition and structure of François' langur and comparison with other primates

Our study identified 134 families and 336 genera of plants in the diet of François' langurs in the Guizhou karst region (Fig. 3), a substantially higher diversity than previously reported. Li recorded 77 families, 182 genera, and 259 plant species across Guangxi and Guizhou Province. Among them, langurs consumed 48 families, 81 genera, and 97 species in Nonggang, Guangxi Province. In Fusui, Guangxi Province, 32 families, 47 genera, 51 species were consumed by langurs. Forty-nine families, 90 genera, and 129 species were recorded in the Mayanghe, Guizhou Province (14), while 8 families, 17 genera, and 18 species in Chongqing city (36). The reason for this difference may be related to the method of diet analysis. In this study, metabarcoding technology by collected feces was able to identify many plants that could not be observed by tracking. Because the tracking observation distance is far, once the langurs are disturbed, it is easy to lose the tracking langurs, resulting in incomplete sampling. DNA metabarcoding can capture DNA sequences that occur less frequently, meaning that less frequently foraging foods can also be quantified (37, 38). Although DNA barcoding analysis provides a more comprehensive approach than direct observation in dietary studies, it also has some drawbacks. For example, it can only identify the species, but not the exact location of the food (39).

Rosaceae, Ranunculaceae, Betulaceae, Rutaceae, Anacardiaceae, and Lauraceae were the main components of the diet of François' langur. Varying degrees of affection for these families have also been observed in primates such as white-headed black langurs (40), Sichuan snub-nosed monkeys (41), Guizhou snub-nosed monkeys, and Tibetan macaques (42). *Prunus*, *Betula*, *Citrus*, *Diospyros*, *Euonymus*, *Pittosporum*, *Nauclea*, *Jasminum*, *Zanthoxylum,* and *Cocculus* were common dominant genera in MYH and DSH. This is similar to the results of previous studies on the dietary habits of langurs (14). *Prunus, Citrus,* and *Diospyros* are popular with wild animals because they are tender and juicy and have good flavor and high vitamin C (43, 44). The nonsignificant differences in dietary composition and diversity among different François' langur groups within the same geographic region demonstrate that this species maintains stable feeding adaptation strategies within its specific habitat. The high degree of consistency in food resource selection across distinct groups reflects a conservative foraging strategy shaped by long-term evolutionary processes.

In the MYH region, the dietary inclusion of cultivated plants, such as Citrus, by François' langurs is notably higher than in the DSH region (Fig. 4). This disparity may be attributed to the practice of supplemental feeding in certain areas of MYH. LEfSe analysis reveals that the family Ulmaceae significantly contributes to the observed differences between the MYH and DSH regions (Fig. 5). Previous studies have corroborated that Ulmaceae species constitute a major component of François' langurs' diet in the Mayanghe region (14), whereas François' langurs in other reserves exhibit less frequent consumption of these plants. This variation is likely a result of differing vegetation compositions across habitats. Although François' langurs are specialized to karst habitats, the karst landforms and associated vegetation structures exhibit considerable variation among different geographic populations. In this study, the habitat similarity between different geographic populations was higher than that between different langur groups within the same region (Table 1), whereas the difference in dietary composition between regions was greater than that between different groups within the same region (Fig. 2). This suggests that the variation in the dietary composition of François' langurs may not be driven by habitat differences. Despite substantial variation in plant composition across habitats within the same region, the dietary composition of different groups in this region does not exhibit significant differences (Fig. 2). This phenomenon may be attributed to the langurs' preference for specific plant families or genera, or it may result from incomplete coverage of their foraging areas in the vegetation quadrat surveys. The pronounced differences in dietary composition among geographically distinct populations underscore the behavioral flexibility of François' langurs in adapting to diverse habitats.

### Gut microbes of François' langur and comparison with other primates

Our study identified Firmicutes, Actinobacteria, and Proteobacteria as the dominant phyla in François' langurs (Fig. 7). This differs from Sun’s findings of Firmicutes and Bacteroidetes dominance in MYH populations (45) and the reported 75.38% ± 6.61% Firmicutes dominance in Guangxi populations (46). The reasons for this difference may be related to different sequencing methods, sampling quantities, and geographical differences.

The gut microbiota of François' langurs are characterized by a high proportion of Firmicutes, similar to that of most primates, such as white-headed black langur (*Trachypithecus leucocephalus*) (47), wild rhesus macaques (*Macaca mulatta*) (48), wild western lowland gorillas (*Gorilla gorilla gorilla*) (49), Sichuan Snub-Nosed Monkeys (*Rhinopithecus roxellana*) (50), Guizhou snub-nosed monkeys (*Rhinopithecus brelichi*) (51), and maintaining a high abundance of Firmicutes is thought to be a digestive strategy that evolved in a positive response (52). Langurs foraging large amounts of leaves, which are rich in lignin and cellulose, both of which are difficult to digest. Firmicutes can not only degrade dietary fiber into small molecules, such as butyrate, to allow absorption by the host (53) but also regulate T cells to improve host immunity, prevent intestinal inflammation, and maintain intestinal microbial ecological balance (54).

Actinobacteria was the second largest phylum of François' langur in our study, and there was significantly different between MYH and DSH (*P* = 0.0009) (Fig. 8). While it accounted for only a small proportion in primates such as white-headed black langurs (47) and rhesus monkeys (46, 48). Actinobacteria significantly mediate the decomposition of recalcitrant organic compounds (particularly cellulose and chitin), driving biogeochemical processes in organic matter turnover and global carbon cycling, as well as constituting essential agents in humification. These functional divergences are likely attributable to habitat-specific adaptations among different phylogenetic lineages.

Amino acid biosynthesis, carbohydrate metabolism, and metabolism of cofactors and vitamins were the main metabolic pathways of François' langur (Fig. 10). It is particularly important to have amino acid biosynthesis during development and stress response (55), especially during nutritional stress conditions (56). Besides, environments and nutrients often influence the fatty acid biosynthesis and lipid accumulation (57). Leaf-eating primates generally have a longer digestive tract, which increases food retention time, promotes the breakdown of fiber and secondary metabolites, and the gut microbiota tends to be enriched in pathways related to amino acid production. Differences in the arachidonic acid metabolism pathway may suggest that the gut microbiota of François' langurs in different regions have evolved distinct strategies to help the host cope with specific chemical compounds in plants (such as phytohormones), which could be a manifestation of their dietary plasticity at a microscopic level. The arachidonic acid metabolism pathway serves as a core interface for host-microbe immune interaction. Variations in its abundance may be influenced by two main factors: first, differences in the lipid composition of their diet, and second, varying environmental pressures in different habitats (such as pathogen load and the degree of anthropogenic disturbance). The significant divergence in this pathway implies that the gut microbiota of François' langur populations in DSH and MYH may have undergone functional differentiation, aiding the host in regulating immune balance and responding to localized environmental challenges.

### Correlations between the gut microbiota and diet composition of François' langur in different wild populations

The microbiota is essential for the extraction of energy and nutrition from plant-based diets and may have facilitated primate adaptation to new dietary niches in response to rapid environmental shifts. Multiple studies have demonstrated that habitat-caused changes in diet and surroundings have a marked effect on the gut microbiota (22–24, 58). In our study, we observed significant correlations between dietary composition and gut microbiota using Spearman correlation analysis. *Nectandra, Toddalia*, *Rosa,* and *Carpinus* had the strongest correlation with Firmicutes and Proteobacteria (Fig. 11). These plants potentially harbor distinct compounds, including fibers, polysaccharides, and secondary metabolites, which may selectively enhance the proliferation of microbial communities associated with these bacterial phyla. This observation implies that the regular consumption of such plants by François' langurs could contribute to sustaining a particular gut microecosystem, predominantly characterized by the prevalence of Firmicutes and Proteobacteria.

### Conclusion

This study elucidated the structural and functional variations in the gut microbiota of François' langurs and their associations with dietary habits using metabarcoding and 16S rRNA sequencing. Significant differences in plant diversity with habitats were observed both between regions and among groups within the same region. Notably, however, the dietary composition and gut microbiota of François' langurs demonstrated considerable similarity among groups in the same region, indicating their ability to adapt to environmental variability through dietary flexibility. These findings underscore substantial disparities in both dietary structure and gut microbial composition across populations inhabiting distinct karst ecosystems in Guizhou, China, thereby highlighting the critical role of diet in facilitating host adaptation to local environmental conditions.

## Data Availability

Raw data used in the study were uploaded to the NCBI SRA under accession no. PRJNA1125922 and PRJNA1125110.

## SUPPLEMENTAL MATERIAL

Supplemental tables msphere.00594-25-s0001.xlsx Tables S1-S3.

ASM does not own the copyrights to Supplemental Material that may be linked to, or accessed through, an article. The authors have granted ASM a non-exclusive, world-wide license to publish the Supplemental Material files. Please contact the corresponding author directly for reuse.
